# Does BRCA Mutation Status Influence Ovarian Cancer Onset Timing and Patients’ Treatment Outcomes?

**DOI:** 10.3390/genes16080883

**Published:** 2025-07-27

**Authors:** Kaja Michalczyk, Agata Mokrzycka, Marianna Rudzińska, Barbara Michalczyk, Janusz Menkiszak, Anita Chudecka-Głaz

**Affiliations:** 1 Department of Gynecological Surgery and Gynecological Oncology of Adults and Adolescents, Pomeranian Medical University, Powstancow Wielkopolskich 72, 70-111 Szczecin, Polandnbz@list.pl (J.M.);; 2 Department of Neonatology and Neonatal Intensive Care Subunit, ul. Dekerta 1, 66-400 Gorzow Wielkopolski, Poland

**Keywords:** *BRCA1*, *BRCA2*, ovarian cancer, HGSOC

## Abstract

Background/Objectives: Mutations in the *BRCA1* and *BRCA2* genes are well-known risk factors for ovarian cancer. They are also associated with response to platinum-based chemotherapy; however, their definitive impact on patient prognosis remains not fully understood. This study aimed to investigate the influence of *BRCA* mutation status on the age of ovarian cancer onset and on treatment outcomes in patients with high-grade serous ovarian cancer. Methods: This single-center retrospective analysis included newly diagnosed FIGO stage III and IV HGSOC patients treated between June 2018 and April 2023. Patients’ age, tumor histology, CA125 levels, *BRCA* mutation status, type of treatment (neoadjuvant or adjuvant chemotherapy), and surgical outcomes were collected and analyzed. Survival analyses were performed using the Kaplan–Meier method and log-rank test. Results: Pathogenic mutations were identified in 25 patients (15 in *BRCA1*, 10 in *BRCA2*). Patients with a *BRCA* mutation were diagnosed at a significantly younger age (median 58.78 years) compared to non-carriers (66.81 years; *p* < 0.001), with *BRCA1* carriers being diagnosed the youngest (median 46.52 years). The study found no statistically significant difference in progression-free survival (PFS) between *BRCA* carriers and non-carriers. However, a significant improvement in overall survival (OS) was observed for patients with a *BRCA1* mutation (*p* = 0.036). No significant OS difference was found for *BRCA2* carriers. Conclusions: *BRCA* mutations, particularly in the *BRCA1* gene, are associated with an earlier onset ovarian cancer. *BRCA1* mutation appears to be a favorable prognostic factor for overall survival in patients with HGSOC. Our findings demonstrate the clinical implications of different *BRCA* mutations and support the need for further research in larger cohorts to confirm their influence on prognostic effects.

## 1. Introduction

Despite modern treatment methods, ovarian cancer remains the leading cause of death in women with gynecological cancer. The majority of patients are diagnosed at advanced stages when the cancer has already metastasized, which contributes to its high mortality rate [1,2]. *BRCA1* and *BRCA2* mutations were identified as ovarian cancer risk factors. Patients diagnosed with a *BRCA1* mutation are estimated to have a lifetime risk of ovarian cancer equal to 40–60% by the age of 80. In comparison, in the *BRCA2* population, the lifetime risk is estimated to be between 11 and 27% [3,4,5]. Multiple prognostic factors were determined that impact patients’ prognosis during ovarian cancer treatment, including patient age, FIGO staging [6], the extent of residual disease after cytoreductive surgical treatment [7,8], tumor histology and grading [6], ECOG status, and response to platinum-based chemotherapy.

Several predictive factors were also selected to determine the groups of patients likely to benefit from particular therapies. Homologous recombination deficiency is the leading predictive factor associated with response to platinum-based chemotherapy and maintenance treatment with PARP inhibitors. *BRCA1* and *BRCA2* mutations were also found to be associated with an improved response to platinum-based chemotherapy [6,9,10]. However, there is still conflicting evidence regarding the association between BRCA mutation and a patient’s prognosis. While studies demonstrate the association between BRCA mutation, response to platinum-based chemotherapy, and progression-free survival, the specific impact of a *BRCA* mutation may vary depending on additional risk factors such as tumor histology, staging, and the treatment regimens used as a part of the therapy [11,12,13,14,15,16]

In this study, we aim to investigate the differences in patient prognosis with regard to BRCA mutation status and its influence on the age of disease onset.

## 2. Materials and Methods

This retrospective analysis involved 123 patients newly diagnosed with high-grade serous carcinoma of the ovary (HGSOC), fallopian tube, or primary peritoneum, classified as FIGO stage III or IV. For all patients, first-line surgical and chemotherapy treatment was performed in the Department of Oncological Gynecology between June 2018 and April 2023. The final follow-up was completed at the end of February 2024. Individuals with missing or incomplete clinicopathological information were excluded from the study. Collected data included patient age, tumor histology, CA125 levels at diagnosis, KELIM score (CA-125 Elimination Rate Constant K), ***BRCA*** mutation status (assessed using Next Generation Sequencing via Illumina Miseq), type of chemotherapy (neoadjuvant or adjuvant), type of surgery (primary cytoreductive surgery or interval cytoreductive surgery), and surgical outcomes (optimal, suboptimal cytoreduction, or no surgery). The alternations in the ***BRCA1*** or ***BRCA2*** genes were classified as pathogenic according to the ClinVar report.

## 3. Results

### 3.1. Population Characteristics

In the study group, 51 patients (41.5%) received neoadjuvant chemotherapy, while the remaining 72 patients (58.5%) were treated with adjuvant therapy. Most of the patients (101 individuals) were diagnosed with stage III disease according to the FIGO classification, and 22 were identified as having stage IV disease. Next-generation sequencing (NGS) did not detect pathogenic *BRCA* mutations in most of the participants. However, 15 patients carried a pathogenic *BRCA1* mutation, and 10 had a pathogenic *BRCA2* mutation. One patient had a previous history of breast cancer. The average age at the time of diagnosis was around 63 years, although there was a broad age range from 25 to nearly 84 years. In the entire cohort, the mean progression-free survival was 1.77 years, and the mean overall survival was 2.33 years (Table 1).

In the group of patients diagnosed with a *BRCA1* mutation, the most common mutation variant was nonsense (seven patients). Four patients were found to have a frameshift mutation, three had missense mutations, and one had a splice acceptor mutation. Among *BRCA2* mutations, the most common was a frameshift mutation (five patients), while four patients had a missense mutation and one had a nonsense mutation.

The distribution of qualitative demographic variables was compared between the patients identified as having pathogenic *BRCA1* and/or *BRCA2* mutations vs. those in which the mutation was not found. There was a significant difference in the age of the patients at the time of ovarian cancer diagnosis. Patients with a *BRCA* mutation were diagnosed at a significantly younger age than the non-*BRCA*-mutated population. The study did not show significant differences between the treatment groups for CA125 at diagnosis and the KELIM constant (Table 2).

When calculated separately for *BRCA1* and *BRCA2* mutations, there were also significant differences in patients’ age at diagnosis onset, with a median age of 46.52 for the *BRCA1* population and 63.83 for *BRCA2* patients (*p* < 0.001). Using a multiple comparisons test, the differences were statistically significant between the *BRCA1* and non-*BRCA*-mutated populations (mean rank difference [D] = −38.30; *p* = 0.016), as well as for the *BRCA1* and *BRCA2* populations (D = −39.84; *p* < 0.001). There were no significant differences between the *BRCA1* and *BRCA2* patient groups (D = −1.54, *p* = 0.456).

### 3.2. Comparison of BRCA Mutation vs. Non-BRCA Population

The distribution of qualitative demographic variables was compared between patients with *BRCA1* or *BRCA2* mutations and *non-BRCA* carriers. Variables such as type of surgery (ICS/PCS), FIGO stage, presence of tumor residues after cytoreductive surgery (R0 or <1 cm vs. >1 cm), ascites, and hydrothorax were evaluated. There were no significant differences in the assessed variables between the different groups of patients (Table 3).

### 3.3. Survival Analysis

Survival analyses were performed using Kaplan–Meier analysis and the log-rank test. Two main time variables, progression-free and overall survival, were analyzed and expressed in years. The log-rank test was used to compare the curves between the assessed groups and, where appropriate, point survival estimates (e.g., 4- and 5-year) were created.

**Figure 1:**
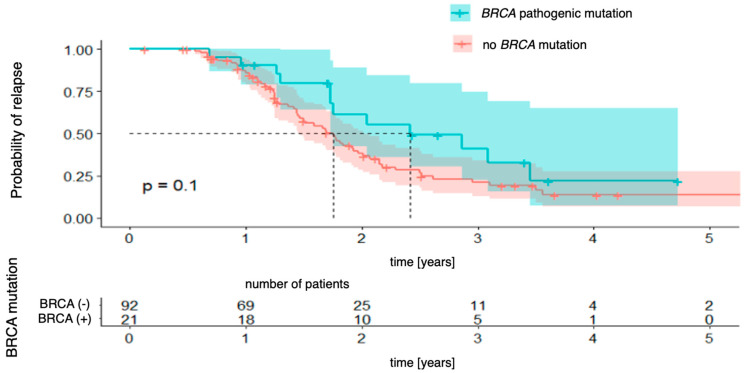
Progression-free survival stratified by *BRCA* mutation status.

Figure 1 demonstrates the relationship between progression-free survival and BRCA mutation status (regardless of mutation type). The analysis did not reveal any statistically significant differences between patients with and without the mutation (χ2 (1) = 2.65; *p* = 0.103), although there was a trend towards a more favorable prognosis in the group with the mutation. The 3-year probability of maintaining a progression-free status was 22% (95% CI [7.3–65.1%]) in the group with the mutation and 14% (95% CI [6.8–27.4%]) in the group without the mutation; however, the differences did not reach statistical significance.

**Figure 2:**
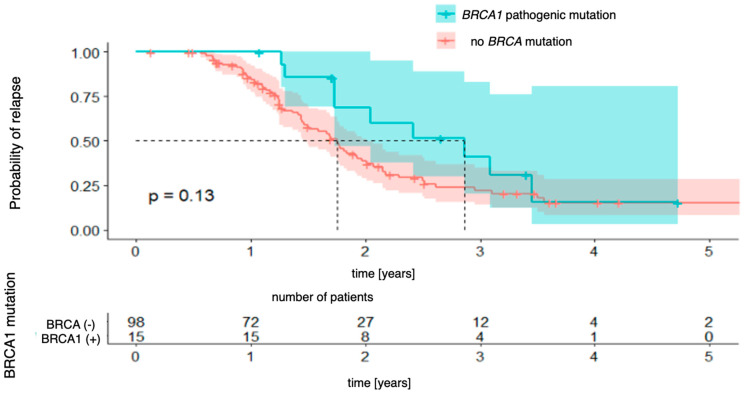
Progression-free survival for *BRCA1*-mutation patients vs. patients with no pathogenic *BRCA* mutation.

**Figure 3:**
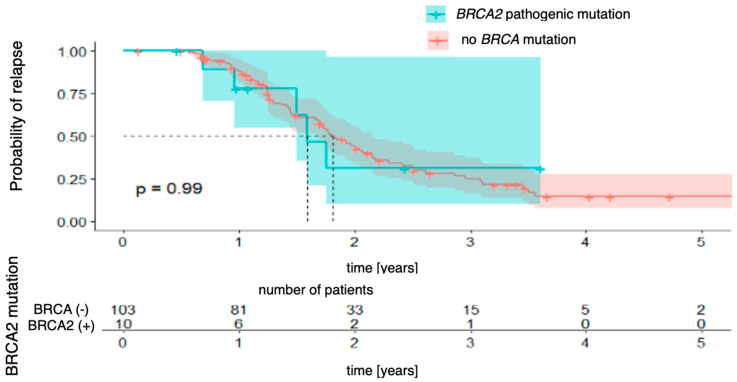
Progression-free survival for *BRCA2*-mutation patients vs. patients with no pathogenic *BRCA* mutation.

Next, the Kaplan–Meier curves were created for progression-free survival concerning the presence of *BRCA1* and *BRCA2* mutations, which were analyzed separately. For *BRCA1* mutations (Figure 2), the analysis did not reveal any statistically significant differences between the groups (χ2 (1) = 2.30; *p* = 0.130). The 3-year probability of maintaining a progression-free state was almost identical in both groups, being 15% (95% CI [8.2–28.6%]) in the group without the mutation and 15% (95% CI [3.0–80.6%]) in the group with the mutation, with the vast confidence interval in the latter indicating a considerable uncertainty in the estimate. For *BRCA2* mutations (Figure 3), there was also no significant difference (χ2 (1) = 0.01; *p* = 0.990). The 3-year progression-free survival was 25% (95% CI [16.7–37.2%]) in the wild-type group and 31% (95% CI [10.1–96.2%]) in the mutation-positive group.

**Figure 4:**
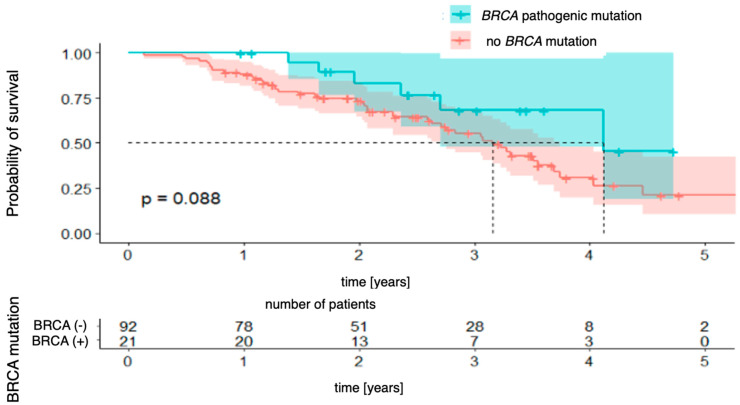
Overall survival stratified by *BRCA* mutation status.

Subsequent Kaplan–Meier survival curves were generated for *BRCA* mutation status (*BRCA* pathogenic mutation vs. no *BRCA* pathogenic mutation). The analysis did not reveal a statistically significant difference in overall survival between patients with and without the mutation [χ2 (1) = 1.92; *p* = 0.088]. However, there was a clear trend toward a better prognosis in the mutation-positive group. The 4-year survival probability in this group was 68% (95% CI [48.0–96.8%]), whereas in patients without the mutation, it was estimated at 31% (95% CI [19.7–48.0%]). This difference did not reach statistical significance, but the divergence of the curves suggests that *BRCA* mutation status may be associated with better long-term survival (Figure 4).

**Figure 5:**
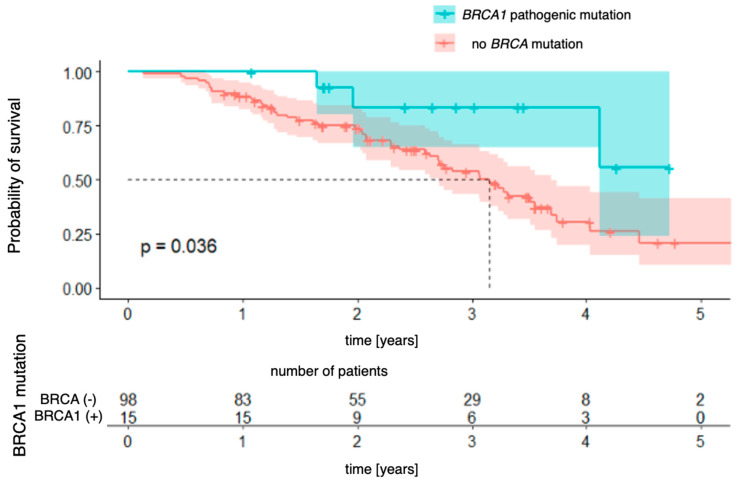
Overall survival for *BRCA1*-mutation patients vs. patients with no pathogenic *BRCA* mutation.

**Figure 6:**
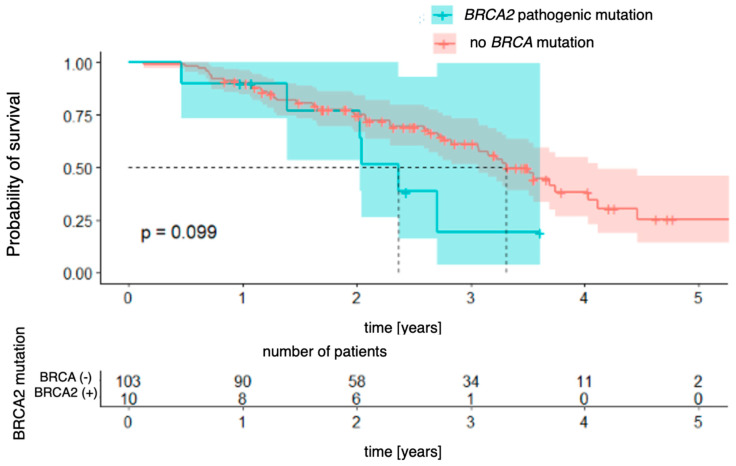
Overall survival for *BRCA2*-mutation patients vs. patients with no pathogenic *BRCA* mutation.

The relationships with the occurrence of *BRCA1* and *BRCA2* mutations were analyzed successively. In the case of the *BRCA1* mutation (Figure 5), a significant difference in overall survival was observed between patients with and without the mutation [χ2 (1) = 4.41; *p* = 0.036]. The four-year probability of survival in patients with a *BRCA1* mutation was as high as 84% (95% CI [64.9–100.0%]), while in the group without a mutation, it was estimated at 30% (95% CI [19.6–47.2%]). For the *BRCA2* mutation (Figure 6), the differences did not reach the level of statistical significance [χ^2^(1) = 2.73; *p* = 0.099], but the direction of the effect indicated a worse prognosis in patients with this mutation.

## 4. Discussion

***BRCA*** mutations significantly increase the risk of ovarian cancer, and the cumulative risk increases with a patient’s age. Even though the age of cancer diagnosis varies, patients with ***BRCA*** mutations seem to have a lower age of cancer diagnosis when compared to the general population of patients, with the mean age of cancer diagnosis being around 51 years for ***BRCA1*** patients and 61 years for ***BRCA2*** patients [17]. In our study, patients with a ***BRCA*** mutation were diagnosed at a significantly younger age when compared to the non-***BRCA***-mutated population (with a median of 58.78 years for ***BRCA1/2*** and 66.81 for non-***BRCA*** patients). When calculated separately for ***BRCA1*** and ***BRCA2*** mutations, there were also significant differences in patient age at diagnosis, with a median age of 46.52 for the ***BRCA1*** population and 63.83 for ***BRCA2*** patients. The results are similar to those in the literature; however, several factors may influence a patient’s age at diagnosis, including ***BRCA*** mutation type (e.g., nonsense, frameshift) [17], family history of breast or ovarian cancer [18,19], or exposure to certain environmental factors [20]. Although the ***BRCA1*** and ***BRCA2*** genes have been sequenced in millions of women, some of the mutation variants still cause difficulties in their clinical interpretation. The problem is driven by the large number of variants of uncertain significance (VUS), as there are hundreds of possible single-nucleotide variants (SNVs) that have received conflicting interpretations [21]. A study on saturation genome editing was performed to accurately classify BRCA1 variants in 13 exons encoding functionally critical domains of ***BRCA1***. The study results showed an almost perfectly concordant distribution of functional effects for almost 4000 SNVs with the previously established assessments of pathogenicity [22]. A similar large-scale saturation mutagenesis evaluation was performed for the ***BRCA2*** gene, generating functional scores for 6551 SNVs [23]. Sequencing maps serve as valuable resources for interpreting previously unidentified variants of ***BRCA*** mutations and provide additional knowledge to the ClinVar data, allowing easier interpretation regarding the pathogenic/non-pathogenic variants of ***BRCA1/2*** mutations and allowing us to better predict the overall survival of individual patients.

The literature shows conflicting data regarding the influence of ***BRCA*** mutation on patient prognosis; moreover, the relative prognoses of ***BRCA1/2*** carriers remains unclear. Sims et al. showed patients with ***BRCA***/HRD− tumors to have worse PFS and OS when compared to germline ***BRCA***+ or somatic ***BRCA***/HRD+ status [24]. A favorable prognosis among ***BRCA*** carriers was also demonstrated by Chetrit et al. [25], Cass et al. [9], and Boyd et al. [26]. On the other hand, Yang et al. demonstrated a more favorable treatment outcome only for ***BRCA2*** mutation carriers and no significant difference for ***BRCA1*** mutation carriers when compared to non-carriers [27].

Several studies have shown no influence of ***BRCA*** mutation on treatment outcomes. A study by Liontos et al. showed no influence of ***BRCA1/2*** mutation (even considering the different locations of the genes) on survival outcomes in HGSOC patients regarding PFS and OS. The authors suggested the potential effect of co-contributing risk factors and other genetic abnormalities to further influence patient prognosis [28]. There are higher rates of concurrent ***TP53*** mutations among patients with ***BRCA*** germline or somatic mutations. ***TP53*** mutations were associated with primary platinum sensitivity in high-grade serous cancer patients, even when adjusted for covariates such as ***BRCA*** mutation status [29]. In our study, we only stratified the patients for ***BRCA*** mutation status and did not consider the presence of any other concurrent mutations. The lack of survival differences was also demonstrated in the studies conducted by Lee et al. [30], Buller et al. [31], and the United Kingdom Coordinating Committee for Cancer Research (UKCCCR) Familial Ovarian Cancer Study Group [32].

A ten-year survival analysis showed that despite the initial survival advantage of patients with a BRCA mutation, there is no difference in long-term survival. The authors suggested the possible correlation of ***BRCA*** mutation with a higher initial sensitivity of BRCA carriers to chemotherapy. However, they found that the surgical status of no residual disease was the strongest predictor of long-term survival [33]. The same group of researchers demonstrated a short-term survival advantage to be associated with carrying inherited ***BRCA1*** or ***BRCA2*** mutations and that there was a lower annual mortality rate during the first two years post-diagnosis among mutation carriers compared to non-carriers, with the trend reversed from year three onwards [34]. Also, a retrospective analysis of the multicenter MITO trial showed R = 0 status as the only predictor of longer overall survival [35].

Our study did not reveal any statistically significant differences in PFS between patients with and without the ***BRCA*** mutation. There were also no differences in separate analyses differentiating for ***BRCA1*** and ***BRCA2*** mutations compared to the non-***BRCA*** population. As for overall survival, the study did not reveal a statistically significant difference in overall survival between patients with and without the mutation. Upon separate analysis, in the case of the ***BRCA1*** mutation, a significant difference in overall survival was observed between patients with and without the mutation (*p* = 0.036). The four-year probability of survival in patients with a ***BRCA1*** mutation was as high as 84% (95% CI [64.9–100.0%]), while in the group without a mutation, it was estimated at 30% (95% CI [19.6–47.2%]). For the ***BRCA2*** mutation, the differences did not reach statistical significance.

The strength of our study was the homogenous distribution of patient characteristics and prognostic variables among the different groups. Our results show no differences between ***BRCA*** and non-***BRCA*** populations concerning the type of performed surgery (ICS/PCS), FIGO stage, presence of tumor residues after cytoreductive surgery (<1 vs. >1 cm), ascites, and hydrothorax. However, our study had some limitations, including the retrospective design of the study and the relatively limited sample size of the ***BRCA***-mutated population. Moreover, the database included only patients diagnosed with high-grade serous ovarian cancer. This allowed us to perform survival analysis on a relatively homogenous population of patients. On the other hand, it might have affected the analysis regarding patients’ age at ovarian cancer diagnosis as, for example, ***BRCA*** mutation carriers are sometimes incidentally diagnosed at the time of prophylactic surgery. During the study, our center did not have the ability to perform HRD testing for the non-BRCA population of patients. As HRD status significantly influences patient prognosis, the survival outcomes might have been different for the HRD and HRP patients that we were unable to categorize. Also, the survival outcome might have been influenced by the maintenance therapy and subsequent therapies used as a part of ovarian cancer treatment. At the time of the study, only BRCAm patients were able to receive maintenance Olaparib treatment, and on 1 January 2022, niraparib treatment was introduced a first-line treatment for advanced ovarian cancer patients regardless of BRCA mutation status. Among the studied population, 20 patients received niraparib maintenance, 10 olaparib monotherapy, and 5 patients underwent combined olaparib plus bevacizumab treatment.

Despite the literature data showing a superior response to platinum-based chemotherapy and a potential initial short-term survival benefit among the ***BRCA***-mutated population of patients, there is no clear answer if the results translate to long-term survival [10,34,36]. Further long-term survival studies are needed, taking into consideration other prognostic factors such as tumor histology, staging, ***BRCA*** and HRD status, and the chemotherapy scheme used, as well as the residual disease after cytoreductive surgery. HRD testing, especially among the ***BRCA1/2*** negative population, seems to be an important predictor for patients’ treatment response and overall survival and a strong prognostic marker, independent of its role in predicting PARP inhibitor sensitivity [37]. While ***BRCA1*** and ***BRCA2*** mutations are the most common cause of homologous recombination deficiency, HRD can also be caused by mutations in different homologous recombination repair (HRR) pathway genes including ***ATM***, ***CHECK2***, ***PALB2,*** and ***RAD51C*** [38] or epigenetic silencing of HRR genes including promoter methylation of the ***BRCA1*** gene [39]. ***BRCA1*** promoter methylation is reported to occur in approximately 10-15% of high-grade serous ovarian cancer tumors [39,40,41,42]. As ***BRCA1***-methylated ovarian cancer shows similar clinicopathological characteristics to ***BRCA1***m ovarian cancer, researchers have begun investigations on its influence on patient survival. A recent meta-analysis of ***BRCA1*** promoter methylation showed no survival differences between ***BRCA1***-methylated and non-***BRCA***-methylated ovarian cancer [39]. However, there is still limited data regarding the impact of methylation status on patient prognosis.

## 5. Conclusions

This study provides an additional basis for further research using larger patient series. It underlines the different aspects of germline ***BRCA1/2*** mutations not only on the age of disease onset but also on patient prognosis. Our results suggest the possible influence of ***BRCA1*** mutation status on overall patients survival.

## Figures and Tables

**Table 1:** Descriptive statistics of quantitative research variables (N = 123).

	R	M	SD	Mdn	Q1–Q3	Sk	Kurt	D
age at diagnosis	25.21–83.97	63.48	11.24	65.61	58.78–70.41	−0.88	0.77	0.12 **
progression-free time [y]	0.4–5.43	1.77	1.04	1.47	1.03–2.17	1.30	1.60	0.13 **
total survival time [y]	0.14–5.43	2.33	1.17	2.23	1.26–3.28	0.39	−0.55	0.09 *
KELIM	0.27–2.20	0.97	0.40	0.94	0.69–1.10	0.91	0.98	0.15 **
CA125	6.20–21534.00	982.62	2368.41	249.50	42.50–1034.75	6.33	50.09	0.34 **

* *p* < 0.05; ** *p* < 0.01.

**Table 2:** Comparison of the assessed variables between patients with *BRCA1* and/or *BRCA2* mutation vs. no *BRCA* mutation.

	*BRCA* Mutation		No *BRCA* Mutation			
	Mdn	SD	Mdn	SD	U	*p*
Age at diagnosis	58.78	11.85	66.81	10.80	472.00	<0.001
CA125	845.50	949.61	492.00	2185.17	872.00	0.196
KELIM	0.91	0.48	0.96	0.39	952	0.932

**Table 3:** Comparison of qualitative demographic variables between patients with *BRCA* mutation and the non-*BRCA* population.

		*BRCA1* Mutation		*BRCA2* Mutation		No *BRCA* Mutation			
		N	%	N	%	N	%	*χ* ^2^	*p*
Surgery	PCS ICS	12 4	18.2 8.5	3 3	4.5 6.4	51 40	77.3 85.1	2.20	0.333
FIGO	III IV	15 1	14.0 16.7	5 1	4.7 16.7	87 4	81.3 66.6	1.72	0.423
Residual disease *	R0 and <1 cm >1 cm	12 4	15.8 11.8	5 1	6.6 2.9	59 29	77.6 85.3	1.00	0.605
Ascites	Yes no	11 5	18.3 9.4	4 2	6.7 3.8	45 46	75.0 86.8	2.50	0.286
Hydrothorax	Yes no	4 12	16.0 13.6	1 5	4.0 5.7	20 71	80.0 80.7	0.18	0.913

* residual disease < 1 cm included R0 population.

## Data Availability

Data available upon request from the corresponding author.
